# Suppressor of Cytokine Signaling 3: Emerging Role Linking Central Insulin Resistance and Alzheimer’s Disease

**DOI:** 10.3389/fnins.2018.00417

**Published:** 2018-06-20

**Authors:** Lan Cao, Zigao Wang, Wenbin Wan

**Affiliations:** ^1^The State Key Laboratory of Medical Neurobiology, The Institutes of Brain Science and the Collaborative Innovation Center for Brain Science, Shanghai Medical College, Fudan University, Shanghai, China; ^2^Department of Neurology, Huashan Hospital, Fudan University, Shanghai, China; ^3^Department of Neurology, Renji Hospital, School of Medicine, Shanghai Jiao Tong University, Shanghai, China

**Keywords:** Alzheimer’s disease, insulin resistance, SOCS3, neuroinflammation, therapeutic target

## Abstract

Currently, the etiology of Alzheimer’s disease (AD) is still elusive. Central insulin resistance has been determined to play an important role in the progress of AD. However, the mechanism underlying the development of disrupted insulin signaling pathways in AD is unclear. Suppressor of cytokine signaling 3 (SOCS3) is a member of the SOCS protein family that acts as a negative modulator of insulin signaling in sensitive tissues, such as hepatocytes and adipocytes. However, little is known about its role in neurological diseases. Recent evidence indicates that the level of SOCS3 is increased in the brains of individuals with AD, especially in areas with amyloid beta deposition, suggesting that SOCS3 may regulate the central insulin signaling pathways in AD. Here, we discuss the potential role of SOCS3 in AD and speculate that SOCS3 may be a promising therapeutic target for the treatment of AD.

## Introduction

Alzheimer’s disease (AD) is a devastating neurodegenerative disease that leads to dementia. It is clinically characterized by the progressive loss of learning and memory and by abnormal mental states and behaviors; pathologically, it is characterized by extracellular amyloid beta (Aβ) deposition and intracellular hyperphosphorylation of tau protein in the brain (Wan et al., 2015, 2016). Extensive efforts have been made to understand the underlying mechanisms of this disease. However, the etiology of AD is still elusive, and we still have no truly effective therapeutic agents to prevent the progression of the disease.

Insulin signaling is activated by circulating insulin and promotes a variety of metabolic pathways, including glucose storage and uptake, protein and lipid synthesis, and mitogenic responses (Chang et al., 2004). Insulin signaling is also critical for cell survival and for the maintenance of physiological functions in multiple tissues (Chang et al., 2004). Thus, disrupted insulin signaling—the most common of which is insulin resistance—can give rise to dysfunctions in both peripheral organs and the central nervous system (CNS). Insulin resistance is a pathological condition involving a failed response to normal levels of insulin. It is a precursor to diabetes mellitus as well as a noteworthy determinant in other metabolic disorders (Soumaya, 2012; Gustafson et al., 2015; Mayans, 2015). Insulin signaling in the CNS is important because of the conspicuous relationship between dysfunctional insulin signaling and disturbed learning and memory in neurodegenerative diseases, such as AD (van der Heide et al., 2006; Chen et al., 2014; Cai et al., 2015). Growing evidence suggests that insulin signaling may be a promising therapeutic target for the treatment of AD. Insulin treatment by intranasal administration has consistently been shown to decrease the production of Aβ and to ameliorate learning and memory deficits in individuals with AD (Reger et al., 2008a,b; Craft et al., 2012; Claxton et al., 2015). However, the molecular mechanisms responsible for the generation of insulin resistance in AD are still unclear.

Suppressor of cytokine signaling 3 (SOCS3) is a member of the SOCS protein family, which was initially characterized by its members’ negative regulatory effects on cytokine signaling, including insulin signaling, in adipocytes (Emanuelli et al., 2000). However, the role of SOCS proteins in the CNS remains unclear. Recently, investigators have reported increased levels of SOCS3 in the brain tissues of individuals with AD (Walker et al., 2015; Cianciulli et al., 2017). Notably, the levels of SOCS3 are correlated with the deposition of Aβ (Walker et al., 2015; Cianciulli et al., 2017; Iwahara et al., 2017), suggesting that SOCS3 might be a novel target that regulates insulin signaling in the brains of AD patients.

In the present review, we first discuss evidence from published studies that have examined the potential role of SOCS3 in AD, suggesting that SOCS3 is a potential target in the treatment of AD.

## Insulin Resistance in AD

Insulin recognizes the α-subunit of the insulin receptor, subsequently leading to autophosphorylation of tyrosine residues and activation of the intracellular β-subunit (IRβ) (Lee and Pilch, 1994). Insulin receptor substrates (IRS) are then tyrosine-phosphorylated, and the downstream insulin signaling pathway is activated (Lee and Pilch, 1994). During a state of insulin resistance, cells exhibit decreased sensitivity to stimulation by insulin, which presents as a reduction in the tyrosine phosphorylation levels of IRβ and IRS and results in insufficient uptake of glucose and amino acids (Boucher et al., 2014). Insulin resistance in neurons also results in many other disturbances, such as synapse dysfunction, disruption of dendritic spines, and disorders of neurotransmitter metabolism (Kleinridders et al., 2014).

Accumulating evidence indicates that insulin resistance plays a critical role in the development of AD (Frame and Zheleva, 2006; Liu et al., 2011; Morris and Burns, 2012; Son et al., 2012; Talbot et al., 2012; Chen and Zhong, 2013; Cai et al., 2015; Mullins et al., 2017). Insulin signaling regulates amyloid precursor protein (APP) metabolism through α-, β-, and γ-secretases. During insulin resistance, the enzyme activities of β- and γ-secretase increase, which subsequently leads to the overproduction of Aβ in neurons (Son et al., 2012). Furthermore, accumulation of Aβ may also enhance the degree of insulin resistance and amplify the damage to the brain (Cai et al., 2015). Aberrant phosphorylation of the tau protein in neurons is another important hallmark of AD. Interestingly, the tau phosphorylation levels are also greatly modulated by insulin signaling (Mullins et al., 2017). In rodent models of insulin resistance, abundant phosphorylation of tau has been identified. More importantly, insulin treatment is able to ameliorate the tau pathology; the activity of glycogen synthase kinase-3 (GSK-3) is considered essential for this effect (Frame and Zheleva, 2006; Chen and Zhong, 2013).

In AD individuals, brain insulin signaling is disturbed, as has been determined from postmortem tissues (Liu et al., 2011; Morris and Burns, 2012). Specifically, the protein levels of the insulin receptor and its substrates are decreased compared to the levels in the brains of individuals without AD (Liu et al., 2011; Morris and Burns, 2012). Talbot and co-workers demonstrated that cultured brain slices from AD patients were insensitive to insulin treatment, revealing the presence of insulin resistance in AD brains (Talbot et al., 2012). Thus, targeting insulin signaling pathways in the brain is a promising therapy for AD. In previous studies, insulin has been employed to treat individuals with AD. These studies found that intranasal insulin treatment delayed memory loss, improved patients’ cognition, and ameliorated the accumulation of plaques in the brain (Reger et al., 2008b; Dhamoon et al., 2009; Stein et al., 2011; Craft et al., 2012). An insulin-signaling sensitizing agent, metformin, has been highlighted as a potential therapeutic agent for AD because of its protective effects against memory impairment and Aβ deposition (Ou et al., 2017). In that study, researchers also found that metformin triggered neurogenesis and presented anti-inflammatory activity in the brains of AD mice (Ou et al., 2017). Although these findings indicate that administration of insulin may be helpful in treating AD, the effectiveness and safety of this treatment require further investigation. Furthermore, contrary evidence still exists, as metformin has also been found to facilitate the generation of Aβ via upregulation of β- and γ-secretases (Chen et al., 2009; Son et al., 2016); however, in these studies, a familial disease model of AD was employed, although sporadic AD is the most common type of the disease. Thus, overall, the details regarding the development of dysfunctional insulin signaling in the brain are presently unclear, and the underlying mechanisms of insulin resistance in AD require further elaboration.

## SOCS3 as a Negative Regulator in Insulin Signaling

The protein members of the SOCS family were first identified in 1997 and have been determined to act as negative modulators of cytokine signaling in various tissues, such as adipocytes, hepatocytes, and tissues of the immune system and CNS (Endo et al., 1997; Naka et al., 1997; Starr et al., 1997). Thus far, eight structurally similar members, including SOCS1-7 and the cytokine-inducible Src homology 2-containing (SH2) protein (CIS), have been characterized in this family (Howard and Flier, 2006). The SOCS proteins comprise three distinct domains: an *N*-terminal domain with a variable length but without a recognizable motif, a well conserved central SH2 domain that binds tyrosine phosphorylation, and a more highly conserved C-terminal domain of ∼40 amino acids known as the “SOCS box” (Bullock et al., 2007). Expression of SOCS proteins is mainly increased by the activation of the signal transducer and activator of transcription (STAT) signaling pathway and partially by the NF-κB pathway, both of which are induced by stimuli that interact with their receptors (Rui et al., 2002; Lebrun and Van Obberghen, 2008). By contacting target elements, such as Janus activated kinase (JAK) or tyrosine-phosphorylated cytokine receptors, SOCS proteins inhibit the progress of phosphorylation and subsequently prevent the activation of STAT-induced transcription factors, thus resulting in negative feedback (Lebrun and Van Obberghen, 2008). Expression of SOCS genes is normally extremely low but increases after exposure to cytokines and hormones, such as lipopolysaccharide (LPS) and insulin (Kazi et al., 2014).

The specificity of each member of the SOCS family is not clear. Previously reviews have concisely reported the characteristic of each one in SCOS (Kile and Alexander, 2001; Larsen and Ropke, 2002; Linossi et al., 2013; Kazi et al., 2014). However, SOCS3 is instrumental, as its kinase inhibitory region (KIR) in the *N*-terminal domain is next to the SH2 domain (Lebrun and Van Obberghen, 2008). The KIR is a functional region that inhibits the kinase activity of JAK and insulin receptors (Lebrun and Van Obberghen, 2008). Very recently, two other recognizable regions, the extended SH2 subdomain (ESS; located between the KIR and SH2 domains) and proline (P)-glutamic acid (E)-serine (S)-threonine (T) (PEST) motif (within the SH2 region), have been identified in SOCS3 (Babon et al., 2006; Lebrun and Van Obberghen, 2008). The ESS is a crucial domain that interacts directly with the nearby tyrosine phosphorylation binding site (Babon et al., 2006). The PEST motif has an important intracellular role in promoting SOCS3 turnover and affecting its degradation pathway (Babon et al., 2006). The functions of these domains suggest that the expression of SOCS3 is finely regulated.

Accumulating evidence has identified SOCS3 as an important negative regulator of the insulin signaling pathway in major insulin-sensitive tissues, such as hepatocytes and adipocytes (Rui et al., 2002; Howard and Flier, 2006; Yang et al., 2010). Insulin stimulation results in the upregulation of SOCS3, which subsequently blocks the insulin signaling pathway (Rui et al., 2002; Howard and Flier, 2006; Yang et al., 2010), suggesting that SOCS3 plays an essential role in regulating insulin sensitivity. Recently, clinical investigations have revealed that individuals with insulin resistance, or with a high risk of insulin resistance, have increased SOCS3 levels (Ghanim et al., 2007, 2009). In genetic analyses, *SOCS3* polymorphisms and epigenetic methylation have also been associated with insulin resistance (Ali et al., 2016; Boyraz et al., 2016). Based on these findings, it is reasonable to speculate that SOCS3 is involved in the development of insulin resistance in the human body and that increased levels of SOCS3 could lead to pathological conditions that disrupt the insulin signaling pathway.

In rodent models of insulin resistance, SOCS3 mRNA expression is robustly upregulated in almost all insulin-sensitive tissues, including hepatic, skeletal, and adipose tissues (Emanuelli et al., 2001; Ueki et al., 2004a,b), revealing a strong and direct relationship between SOCS and insulin resistance. Interestingly, no change in SOCS3 expression is observed in rodent models with a deficiency in TNFα receptors (Uysal et al., 1997), indicating that the expression of SOCS3 is dependent on an inflammatory TNFα signal. Several studies have examined the genetic regulation of SOCS3 in different peripheral organs to determine its role in the generation of insulin resistance; these studies have shown that SOCS3 deletion results in the loss of insulin resistance and in enhanced effects of insulin signaling, as evidenced by increased tyrosine phosphorylation of IRS1 (Torisu et al., 2007; Sachithanandan et al., 2010; Jorgensen et al., 2013). Consistent with such findings, SOCS3 overexpression decreases the tyrosine phosphorylation levels of IRS1 and inhibits the activity of phosphatidylinositol-3 kinase (PI3K), a downstream signaling element of IRS1 (Ueki et al., 2004a; Yang et al., 2012), revealing a pivotal role of SOCS3 in insulin resistance. Furthermore, SOCS3-induced ubiquitin-mediated degradation of IRS1 has been demonstrated to participate in the inhibition of insulin in multiple cell types (Rui et al., 2002). Specifically, SOCS3 binds with IRS1 and promotes its ubiquitination (Rui et al., 2002). An elongin BC-containing ubiquitin ligase is subsequently incorporated, which finally results in the degradation of the IRS1 protein (Rui et al., 2002).

Thus, SOCS3 is critical for the inhibition of the insulin signaling pathway. The aforementioned investigations indicate that suppression of SOCS3 is beneficial for the activation of insulin signaling and suggest that SOCS3 could be a new therapeutic target for diseases in which insulin resistance is involved.

## SOCS3 in the Brain

The expression of SOCS3 in the brain was first discovered in the year 2000; detection of mRNA revealed that SOCS3 is apparently widespread in the brain, including in the hippocampus, the granular layer of the cerebellum, the thalamus and the basal ganglia (Polizzotto et al., 2000; Wang and Campbell, 2002). Under physiological conditions, the SOCS3 transcript achieves its maximal levels in the brain from E14 to postnatal day eight, but it drops to quite a low level in adults (Polizzotto et al., 2000). Furthermore, Mishra et al. reported that upregulation of SOCS3 promoted the differentiation of cultured neural stem cells even if no NGF was added to the medium, indicating that SOCS3 is involved in the development of the CNS.

As in peripheral tissues, expression of SOCS3 in the CNS increases rapidly in response to various stress-related stimuli, such as LPS, IFN, IL1, and IL6 (Wang and Campbell, 2002; Steffensen et al., 2014). Qin and coworkers found that IFNβ-induced expression of SOCS3 in astrocytes was dependent on the activation of STAT3. Disruption of SOCS3 by the administration of IFNβ resulted in a large mass of inflammatory cytokines and enhanced the migration of microglial and T cells (Qin et al., 2008). These results indicate that SOCS3 could act as an immune modulator in the CNS.

The precise roles of SOCS3 in the mature neurons of the CNS are still unclear. Nevertheless, it has been found that SOCS3 acts as a negative regulator of neuronal survival and axon regeneration after neural injury (Smith et al., 2009; Liu et al., 2015). Deletion of SOCS3 facilitates cellular survival and axon regeneration in retinal ganglion cells after optic nerve injury (Smith et al., 2009; Liu et al., 2015). Thus, SOCS3 could be a promising modulator of neural repair in the adult CNS, perhaps guarding against long-distance axon regeneration.

## SOCS3 in AD

Expression of SOCS3 has been well defined in human brains, but changes in SOCS3 expression in AD brains had not been examined until a study by Walker and co-workers (Walker et al., 2015). In this work, the researchers found that expression of SOCS3 in the brains of AD patients was significantly greater than expression in the brains of individuals with mild cognitive impairment (MCI) or the brains of non-demented individuals (Walker et al., 2015). They also observed significant correlations between the SOCS3 mRNA levels and scores for Aβ plaques and neurofibrillary tangles (Walker et al., 2015). In alignment with SOCS3’s aforementioned involvement in CNS inflammation, the level of SOCS3 is consistently increased in Aβ-stimulated human microglia (Walker et al., 2015). Furthermore, expression of SOCS3 is regulated by the JAK/STAT signaling pathway (Lebrun and Van Obberghen, 2008). In fact, the JAK/STAT signal transduction pathway can be activated by exposure to Aβ (Capiralla et al., 2012), indicating a potential role for Aβ in regulating the expression of SOCS3. Recently, Iwahara and colleagues demonstrated that both Aβ-stimulated primary cultured microglia and microglia in a APPswe/PS1dE9 transgenic mouse model of AD expressed SOCS3; they further revealed that SOCS3 was involved in the switch to an activated microglia phenotype (Iwahara et al., 2017), suggesting a potential role for SOCS3 in AD, especially in AD-related neuroinflammation.

However, the effect of SOCS3 on insulin resistance in AD has not been defined. Studies have shown that in addition to glucose metabolism and neural activation, impaired neuronal insulin signaling is also involved in proinflammatory signaling associated with AD (Ferreira et al., 2014). In postmortem human brains and in rodent models of AD, Aβ accumulation has been shown to cause deficiencies in insulin signaling, whereby serine phosphorylation of IRS1 is increased and tyrosine phosphorylation of IRS1 is decreased (Bomfim et al., 2012). Indeed, tyrosine phosphorylation of IRS1 acts as an important proinflammatory signal (Bomfim et al., 2012; Ferreira et al., 2014). Subsequent research has revealed that the neurotoxicity resulting from Aβ inhibits tyrosine phosphorylation-mediated signaling by IRS1 and leads to serine phosphorylation-induced activation of IRS1 via TNFα (Ma et al., 2009; Bomfim et al., 2012; Craft, 2012; Ferreira et al., 2014), a modulator of SOCS3, as described above (Uysal et al., 1997).

**FIGURE 1:**
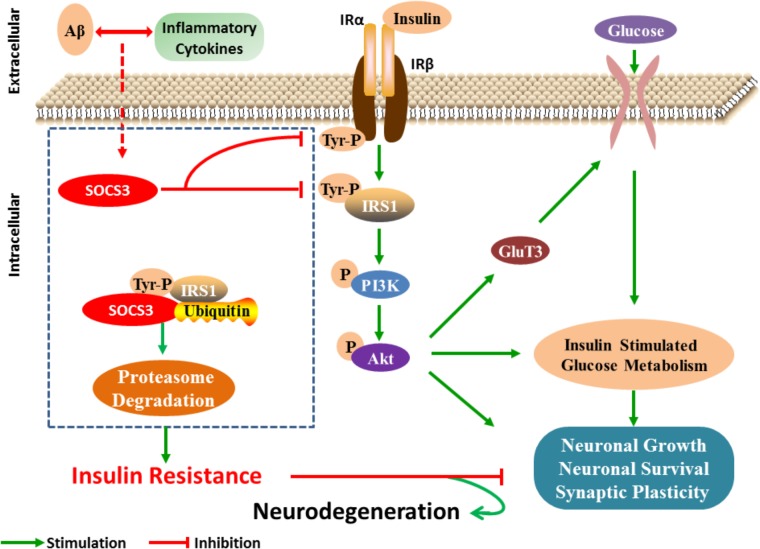
SOCS3 regulates insulin signaling in neuron. After receiving the stimulation of cytokines, the expression of SOCS3 is increased and then insulin signaling is disturbed by inhibiting the tyrosine phosphorylation process of IRS1. Furthermore, serine phosphorylation IRS1 is increased, which then results in the inflammatory effects and neuronal lesions.

Taken together, these studies suggest that SOCS3 is involved in dysfunctional insulin signaling in the brains of patients with AD (**Figure 1**). As SOCS3 has also been identified in peripheral tissues, we speculate that SOCS3 plays an essential role in the establishment of insulin resistance in AD. Further study is warranted to determine the specific relationship between SOCS3 and insulin resistance.

## SOCS3-Modulated Inflammatory Cytokines in AD

As it has been proposed that insulin resistance in diabetes is a consequent result of chronic inflammation leading to the overloaded proinflammatory cytokines (Vieira et al., 2017). The inflammatory mediators are able to cross the blood-brain barrier (BBB) and activate the microglia. Growing evidence has revealed the existence of inflammation in AD brain and peripheral blood (Czirr and Wyss-Coray, 2012; Clarke et al., 2015). It also shows neuroinflammation regulates the impairment of cognition (Heneka and O’Banion, 2007), suggesting that inflammation acts as a linkage between insulin resistance in diabetic and in AD.

In peripheral, activation of inflammatory signal promotes the expression of SOCS3 (Uysal et al., 1997; Yin et al., 2015). In CNS, accumulating evidence revealed the regulatory role of SOCS3 in neuroinflammation. Microglia is the resident immune cells in the brain. Microglia-associated central inflammation plays a deleterious role in neural degeneration resulting in the pathogenesis of AD. Recently, Iwahara and co-workers observed that microglia expressed SOCS3 and exhibited an M1-like phenotype, expression cytokine TNFα but not IL6 in Aβ-stimulated primary culture and APP/PS1 mice (Iwahara et al., 2017). While, elimination of SOCS3 expression resulted in upregulation of IL6 in Aβ-challenged primary microglia (Iwahara et al., 2017), suggesting the role of SOCS3 in suppressing the excessive inflammation induced by M1 microglia. However, the effect of SOCS3 in the rodent AD mice has not been evaluated in this work.

Yet in neuron, the role of SOCS3 presents very different from that in microglia. SOCS3-overexpression inhibited the JAK/STAT3-regulated protective effects of IGF1 against TNFα-induced lesions and led to neuroblastoma cell death, indicating the involvement of SOCS3 in cell survival (Yadav et al., 2005). In primary-cultured sensory neurons, SOCS3 suppressed neurite growth via the inhibition of STAT3 signal, suggesting the detrimental effect of SOCS3 on axonal growth (Miao et al., 2006).

## The Role of SOCS3 in AD

Although many studies have been conducted to explore therapeutic candidates for the prevention and treatment of AD, an effective target has yet to be determined. Previous work has revealed that the Aβ and tau proteins are the two core pathologies in AD brains (Cowan and Mudher, 2013; Wan et al., 2014; Villemagne et al., 2015; Xu et al., 2015). However, in past few decades, multiple treatments, including Aβ immunization (Holmes et al., 2008; Doody et al., 2013) and administration of β-secretase inhibitors (Mikulca et al., 2014), γ-secretase inhibitors (Mikulca et al., 2014; Doody et al., 2016), and tau aggregation inhibitors (Gauthier et al., 2016), have proven to be insufficient to cure AD. Therefore, it is imperative to find more promising targets for disease prevention.

Dysfunctional cerebral glucose metabolism is an early and invariant characteristic of AD (Chen and Zhong, 2013). Some research suggests that dysfunctional neuron metabolism is a critical contributor to AD (Chen and Zhong, 2013). FDG-PET/CT imaging shows consistent and progressive reductions in brain glucose metabolism in individuals with AD, and these reductions are closely correlated with cognitive impairment and clinical severity (Mosconi, 2005). Since insulin signaling is the key factor regulating glycometabolism, it is important to analyse disrupted insulin signaling pathways in AD and to determine the underlying pathogenesis.

As a negative modulator, SOCS3 is a vital switch for the unhindered transduction of insulin signals, which subsequently affects cellular glycometabolism. In peripheral tissues, inhibition of SOCS3 has been proven to activate insulin signaling (Torisu et al., 2007; Sachithanandan et al., 2010; Jorgensen et al., 2013), and expression of SOCS3 is increased in the brains of individuals with AD (Walker et al., 2015; Iwahara et al., 2017). Thus, SOCS3 may also be a promising candidate for ameliorating insulin insensitivity, leading to the improvement of brain glycometabolism and the prevention of disease progression.

**FIGURE 2:**
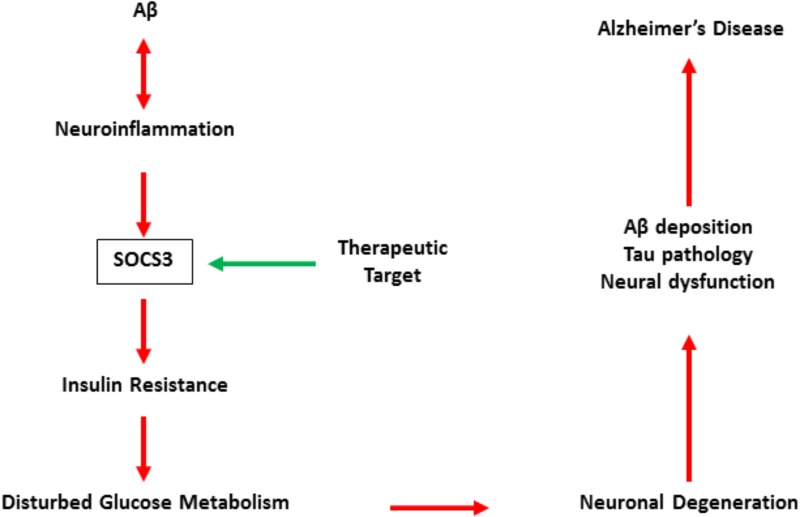
The pattern of SOCS3 in the development of Alzheimer’s disease.

Although direct evidence is still insufficient, relevant studies have revealed that SOCS3 may be a promising target for the treatment of AD (Czirr and Wyss-Coray, 2012; Clarke et al., 2015). SOCS3 is also involved in neroninflammation and is essential for inflammation-regulated insulin resistance in the central (**Figure 2**). Therefore, the accurate role of SOCS3 in AD is still unclear, but its detrimental effects on cell survival and axonal growth indicate SOCS3 as a potential target in neuroral protection (Yadav et al., 2005; Miao et al., 2006).

## Conclusion and Perspective

The regulatory mechanisms of SOCS3 expression in AD are elusive. Aβ deposition and inflammatory factors in the brain are the most important causes of neural lesions (Zhang and Jiang, 2015) and may also initiate upregulation of SOCS3 expression in AD. Cerebral glucose hypometabolism and insulin resistance are the invariant feature of AD and have been revealed to be critical for the development of the disease (Steen et al., 2005; Mosconi et al., 2008; Takeda et al., 2010). Insulin signaling is the most critical modulator of glucose metabolism in various tissues and organs. However, the mechanisms underlying the development of insulin resistance in brains of AD are still unclear. Current evidence as described above does not only indicate that SOCS3-mediated inhibition of insulin signaling is important for the peripheral pathologies in metabolic syndromes, but also suggest the essential role of SOCS3 in the disturbed insulin signaling in the CNS of AD. We speculate that the increased level of SOCS3 resulting from the stimulations of pathological products in brains of AD might lead to the disruption of insulin signaling, which then facilitates the development of AD. Despite all these, further studies are needed to test our hypothesis and to inspect that targeting SOCS3 in the brain would be a promising therapeutic strategy for AD.

## Author Contributions

LC and WW wrote this review. ZW corrected and made the revisions of this review.

## Conflict of Interest Statement

The authors declare that the research was conducted in the absence of any commercial or financial relationships that could be construed as a potential conflict of interest.
